# Radiofrequency thermocoagulation under neuromonitoring guidance and general anesthesia for treatment of refractory trigeminal neuralgia

**DOI:** 10.1007/s00701-024-05964-9

**Published:** 2024-02-02

**Authors:** Tammam Abboud, Vesna Malinova, Veit Rohde, Dorothee Mielke

**Affiliations:** Department of Neurosurgery, University Medical Center Göttingen, Georg-August-University, Robert-Koch-Straße 40, 37075 Göttingen, Germany

**Keywords:** Trigeminal neuralgia, Radiofrequency thermocoagulation, Antidromic sensory–evoked potentials, Neuromonitoring

## Abstract

**Objective:**

Radiofrequency thermocoagulation (RFT) for refractory trigeminal neuralgia is usually performed in awake patients to localize the involved trigeminal branches. It is often a painful experience. Here, we present RFT under neuromonitoring guidance and general anesthesia.

**Method:**

Stimulation of trigeminal branches at the foramen ovale with the tip of the RFT cannula is performed under short general anesthesia. Antidromic sensory–evoked potentials (aSEP) are recorded from the 3 trigeminal branches. The cannula is repositioned until the desired branch can be stimulated and lesioned.

**Conclusion:**

aSEP enable accurate localization of involved trigeminal branches during RFT and allow performing the procedure under general anesthesia.

**Supplementary Information:**

The online version contains supplementary material available at 10.1007/s00701-024-05964-9.

## Background

Trigeminal neuralgia (TN) is the most prominent representative of neuropathic facial pain syndromes [10]. The primary treatment of TN is pharmacotherapy. Guidelines on TN management recommend that patients unresponsive to carbamazepine or oxcarbazepine be offered the surgical option [1] Microvascular decompression (MVD) is the first surgical option if MR-imaging demonstrates a clear neurovascular compression. In other cases, especially after failed MVD, neuroablative procedures become a reliable method that can promise pain relief in 78 to 100% of the cases for a mean time of 8–40 months [9]. Among these options, radiosurgery has become a validated one with a high success rate and low-risk profile [8].

Radiofrequency thermocoagulation (RFT) is a neuroablative procedure that may offer higher rates of complete pain relief than glycerol rhizolysis. Precise positioning of the needle tip during RTF, usually reached by fluoroscopy or CT guidance and a patient’s feedback, is a prerequisite for a successful treatment and reduction of complications. A patient’s feedback allows us to perform a sensory test stimulation confirming the right position of the tip of the cannula and, if needed, to adjust the position and repeat the test stimulation. This maneuver is uncomfortable for most of the patients and can be painful. In addition, verbal responses can be unreliable, which increases morbidity and decreases the chances of treatment success [4]. Neurophysiology-guided positioning of the cannula tip during RFT can offer an alternative to the patient’s feedback and allow the whole procedure to be performed under general anesthesia [5], which is certainly more comfortable for the patient and more convenient for the surgeon. Neurophysiological guidance was described before as a complementary method to the patient’s feedback [4, 5]. We present here the RFT under general anesthesia using neurophysiological monitoring as an alternative to the patient’s verbal responses to confirm the correct position of the cannula and selectively lesion the involved branches of the trigeminal nerve.

## Method

### Relevant surgical anatomy

RFT to treat TN addresses the trigeminal nerve at the level of the Gasserian ganglion, which is situated in Meckel’s cave within the middle cranial fossa. Meckel’s cave is a dura mater pouch containing cerebrospinal fluid and is surrounded by clivus medially, posterior petrous face inferolaterally, cerebellar tentorium superolaterally, and lateral wall of the cavernous sinus superomedially. The three branches of the trigeminal nerve that converge on the Gasserian ganglion are the ophthalmic, maxillary, and mandibular branches. They enter the skull base through three separate foramina that are respectively the superior orbital fissure, the foramen rotundum, and the foramen ovale. The latest is used as an entry point into the skull when performing percutaneous procedures to access the Gasserian ganglion.

### Description of the technique

**Fig. 1:**
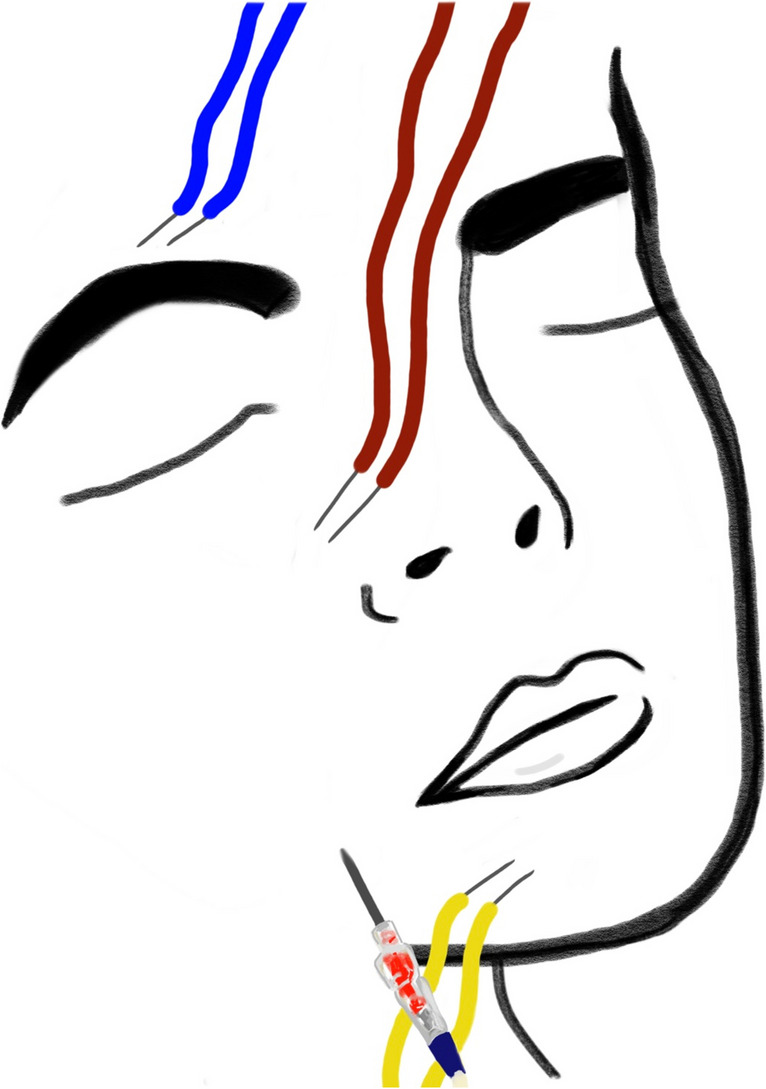
A schematic illustration of intraoperative installation of subdermal needle electrodes to record antidromic sensory evoked–potentials (aSEP) from the different trigeminal divisions (V1, V2, and V3)

**Fig. 2:**
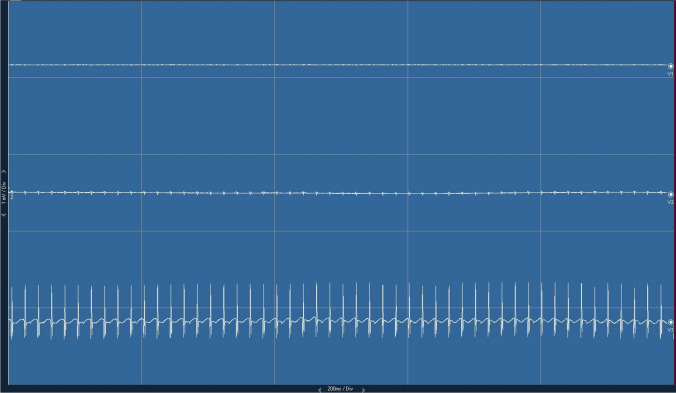
Intraoperative recording of antidromic sensory–evoked potentials (aSEP) from the three trigeminal divisions during RFT in a patient with V3 neuralgia. Electrical stimulation of trigeminal divisions generates aSEP that can be recorded through the subdermal needles implanted in the corresponding facial distribution area of the trigeminal division. The image demonstrates that aSEP were recorded from V3 (third line) after stimulation with the tip of the cannula within Meckel’s cave

Under general anesthesia, induced and maintained by Propofol, the patient is put in the supine position with the head tilted backward. Subdermal needles are implanted into the supraorbital, infraorbital, and mental foramina to record antidromic sensory–evoked potentials (aSEP) from the first, second, and third divisions of the trigeminal nerve (Fig. 1). The NeuroExplorer (inomed Medizintechnik GmbH, Immendingen, Germany) or the Endeavor system (Natus Europe GmbH, Langenfeld, Germany) is used for aSEP recording. RFT is performed in the technique described by Sweet and Wepsic [7] using a 22-gauge, 15-cm radiofrequency cannula with a 5-mm active tip (Boston Scientific Medizintechnik GmbH, Düsseldorf, Germany). The cannula is inserted on the affected face side from a point 2.5 cm lateral and 1 cm caudal to the angular oris and advanced toward the foramen ovale under fluoroscopic guidance. The correct position of the needle tip within Meckel’s cave is confirmed by fluoroscopy and the ability to aspirate cerebrospinal fluid. At this point, electrical stimulation (0.05–0.15 V) is applied at the tip of the cannula generating aSEP from different divisions of the trigeminal nerve. The aSEP correspond to the position of the cannula tip [5]. Electrical stimulation can be repeated, while the cannula is repositioned until the desired position is reached, which is identified by recording the highest possible aSEP from the involved trigeminal division (Fig. 2). Afterward, lesioning of the involved trigeminal division can be undertaken in the conventional way. We recommend lesioning at 70 °C for 90 s. Thereafter, the patient is awakened, a clinical examination is performed, and the patient is transitioned back to the ward. A video demonstrating the intraoperative electrophysiological recording of aSEP confirming the correct position of the tip of the cannula before lesioning was uploaded with the manuscript (Video 1).

### Indications

RFT under neurophysiological guidance is indicated in patients with TN refractory to pharmacotherapy and who have had a failed MVD or in whom MVD is not deemed suitable.

### Limitations

There are no specific limitations to RFT under neurophysiological guidance other than those generally related to RFT. Patients who had previous RFT might harbor arachnoidal adhesions at the level of the Gasseri ganglion that might prohibit aspiration of cerebrospinal fluid as proof of the correct position of the tip of the cannula. However, fluoroscopic and neurophysiological guidance should be sufficient in such cases. Injection of a contrast agent under fluoroscopy can also prove that the cannula tip is located within Meckel’s cave.

### How to avoid complications

Since RFT is a destructive procedure, and it involves a degree of nerve damage that can result in a change in facial sensation. Correct positioning of the RFT cannula should reduce this risk to a minimum. However, patients should be aware of this risk prior to the procedure. In case of loss of the corneal reflex, patients are informed to check their eyes daily for redness and are encouraged to wear glasses when they go outdoors. Performing RFT with high temperature (≥ 75 °C) is associated with a higher risk of serious complications including severe facial numbness, ptosis, keratitis, corneal ulcers, and damage to other cranial nerves [2] and should be therefore strictly avoided. Anesthesia dolorosa (painful numbness) is a rare but severe side-effect that can occur after RFT with a rate of 0.8%, and its incidence is suggested to be reduced through selective lesioning [3]. Cardiovascular complications including bradycardia can occur during the procedure. Therefore, continuous intraoperative monitoring of hemodynamic parameters is essential and can allow the surgeon to interrupt surgical maneuvers immediately upon the occurrence of such complications [6]. In our experience, the administration of atropine may be helpful to prevent repeated bradycardia.

### Specific perioperative considerations

Appropriate patient selection is mandatory to avoid treatment failure and reduce morbidity. Thorough preoperative interpretation of MR-imaging is essential to detect possible compression of trigeminal nerve and brain stem lesions at the level of the trigeminal nucleus, as these findings might change treatment strategy and affect success rates of RFT. The patients should be comprehensively informed of the response rate and risks of RFT as well as alternative therapy options. Instant pain relief after RFT occurs at a rate of 90–100%. However, response rate decreases over time to 58% and 42% after 5 and 11 years respectively [2].

## Summary

Electrophysiology-guided RFT for the treatment of TN can be performed under general anesthesia. aSEP enable accurate localization of involved trigeminal branches before lesioning and thus alleviate the burden of awake procedure for patients with TN.

## Supplementary Information

Below is the link to the electronic supplementary material.

Supplementary file1 Video 1: Electrophysiological assessment of the position of the tip of the cannula during radiofrequency thermocoagulation in a patient suffering from refractory trigeminal neuralgia (V2) under general anesthesia. After entering Meckel’s cave, stimulation with the tip of the cannula results in antidromic sensory–evoked potentials (aSEP) recorded from V2 and V3. Afterward, a slight adjustment of the position of the cannula leads to record significantly higher aSEP from the desired branch (V2). Afterward, selective lesioning can be undertaken. (M4V 2687 KB)

## Data Availability

All available data was presented in the manuscript.
